# Fulvestrant plus palbociclib in advanced or metastatic hormone receptor-positive/human epidermal growth factor receptor 2-negative breast cancer after fulvestrant monotherapy: Japan Breast Cancer Research Group-M07 (FUTURE trial)

**DOI:** 10.1007/s10549-023-06911-5

**Published:** 2023-03-31

**Authors:** Kenichi Watanabe, Naoki Niikura, Yuichiro Kikawa, Mari Oba, Kokoro Kobayashi, Hiroshi Tada, Shinji Ozaki, Uhi Toh, Yutaka Yamamoto, Michiko Tsuneizumi, Toshitaka Okuno, Nobutaka Iwakuma, Takashi Takeshita, Takayuki Iwamoto, Hiroshi Ishiguro, Norikazu Masuda, Shigehira Saji

**Affiliations:** 1 Department of Breast Surgery, Hokkaido Cancer Center, Sapporo, Japan; 2 Department of Breast and Endocrine Surgery, Tokai University School of Medicine, Isehara, Japan; 3 Department of Breast Surgery, Kobe City Medical Center General Hospital, Kobe, Japan; 4 Department of Clinical Data Science, Clinical Research & Education Promotion Division, National Center of Neurology and Psychiatry, Kodaira, Japan; 5 Department of Breast Medical Oncology, Cancer Institute Hospital of JFCR, Tokyo, Japan; 6 Department of Breast and Endocrine Surgical Oncology, Graduate School of Medicine, Tohoku University, Sendai, Japan; 7 Department of Gastrointestinal and Breast Surgery, Hiroshima Prefectural Hospital, Hiroshima, Japan; 8 Department of Breast Surgery, Kurume University Hospital, Kurume, Japan; 9 Department of Breast and Endocrine Surgery, Kumamoto University Hospital, Kumamoto, Japan; 10 Department of Breast Surgery, Shizuoka General Hospital, Shizuoka, Japan; 11 Department of Breast Surgery, Kobe City Nishi-Kobe Medical Center, Kobe, Japan; 12 Department of Breast Surgery, National Hospital Organization Kyushu Medical Center, Fukuoka, Japan; 13 Department of Breast and Endocrine Surgery, Kumamoto City Hospital, Kumamoto, Japan; 14 Department of Breast and Endocrine Surgery, Okayama University Hospital, Okayama, Japan; 15 Breast Oncology Service, Saitama Medical University International Medical Center, Hidaka, Japan; 16 Department of Breast and Endocrine Surgery, Nagoya University Graduate School of Medicine, Nagoya, Japan; 17 Department of Medical Oncology, Fukushima Medical University, Fukushima, Japan

**Keywords:** Advanced breast cancer, Breast neoplasms, CDK 4/6 inhibitors, Selective estrogen receptor modulators

## Abstract

**Purpose:**

The combination of cyclin-dependent kinase 4/6 inhibitors and endocrine therapy is a standard treatment for hormone receptor (HR)-positive/human epidermal growth factor receptor 2 (HER2)-negative metastatic breast cancer (MBC); however, their toxicities and financial burden are major issues, especially for prolonged treatment. We investigated fulvestrant plus palbociclib in patients with HR-positive MBC resistant to fulvestrant monotherapy.

**Methods:**

Patients who initially received fulvestrant as their first- or second-line endocrine therapy were assigned to group A. Patients with disease progression during fulvestrant monotherapy who subsequently received fulvestrant plus palbociclib were assigned to group B. The primary endpoint was progression-free survival (PFS1) in group B. We set the threshold median PFS of 5 months (null hypothesis).

**Results:**

Between January 2018 and February 2020 we enrolled 167 patients in group A (January 2018–February 2020) from 55 institutions, of whom 72 subsequently received fulvestrant plus palbociclib and were enrolled in group B. The median follow-up was 23.8 and 8.9 months in groups A and B, respectively. The median PFS in group B (combination therapy) was 9.4 (90% confidence interval [CI]: 6.9–11.2) months (*p* < 0.001). This was 25.7 (90% CI: 21.2–30.3) months in group A (fulvestrant monotherapy). The TTF in group B was 7.2 (90% CI: 5.5–10.4) months. In the *post-hoc* analysis, the median PFS1 in group B among patients with longer-duration fulvestrant monotherapy (> 1 year) was longer than that of patients with shorter-duration monotherapy (≤ 1 year) (11.3 vs. 7.6 months). No new toxicities were observed.

**Conclusion:**

Our findings suggest that palbociclib plus fulvestrant after disease progression despite fulvestrant monotherapy is potentially safe and effective in patients with HR-positive/HER2-negative advanced MBC.

**Supplementary Information:**

The online version contains supplementary material available at 10.1007/s10549-023-06911-5.

## Introduction

The aim of advanced breast cancer (ABC) treatment is prolonged overall survival (OS) while maintaining the patient’s quality of life (QOL) [1]. ABC treatment has become increasingly complex owing to multiple newly approved drugs for the treatment of hormone receptor (HR)-positive ABC. Fulvestrant, a selective estrogen receptor degrader and standard endocrine therapy (ET) for HR-positive ABC, is associated with prolonged progression-free survival (PFS) in postmenopausal women in both first- and second-line treatment settings [1–4]. The Phase III FALCON trial assessed fulvestrant as a first-line treatment and found significant improvement in the median PFS with fulvestrant than with an aromatase inhibitor (AI) such as anastrozole (16.6 vs. 13.8 months) [3].

Cyclin-dependent kinase (CDK) 4/6 inhibitors are used in ABC treatment, inhibiting CDK 4 and CDK 6 in vitro, resulting in decreased RB1 phosphorylation (a tumor suppressor protein) [5]. Positive activity has been observed in breast cancer cell lines with these inhibitors when used in monotherapy, which is synergistic with other endocrine therapies [6]. In patients with metastatic, estrogen receptor-positive breast cancer with AI resistance in the PALOMA-3 trial, the combination of palbociclib–fulvestrant therapy was associated with significantly longer PFS than that of fulvestrant alone [7, 8]. These findings suggest that palbociclib may potentially reverse ET resistance in patients with previous responses to ET, making other drugs, such as AI or fulvestrant, more effective. The TREnd trial investigated the activity of palbociclib combined with the same ET that was received prior to disease progression. The clinical benefit rate was 54%, and the median PFS was 10.8 months in patients who received combination therapy. This benefit may be attributed to the additional therapeutic action of ET following the reversal of prior ET resistance by palbociclib [9].

Patients with ABC often receive multiple drugs throughout treatment, which can negatively impact their QOL; therefore, the adverse-event profile of any treatment is particularly important in this setting. Hence, less toxic treatment strategies that favor HR-positive patients regarding non-difference in OS are important and remain an unmet clinical need. Furthermore, the addition of palbociclib to fulvestrant therapy costs an estimated $918,166 per quality-adjusted life-year (QALY) gained, which is nine times higher than the willingness-to-pay threshold of $100,000 per QALY [10].

We hypothesized that adding palbociclib to fulvestrant therapy would be effective in patients with HR-positive ABC that has progressed despite fulvestrant therapy. Our primary objective was to observe PFS in patients resistant to fulvestrant monotherapy and subsequently treated with fulvestrant plus palbociclib. The secondary objective was to observe PFS in HR-positive ABC patients treated with fulvestrant as first- and second-line therapy.

## Methods

### Study protocol

The FUTURE trial was a multicenter, prospective cohort study that evaluated the safety and effectiveness of adding palbociclib to fulvestrant therapy for patients with HR-positive ABCs and disease progression despite fulvestrant monotherapy. Patients receiving fulvestrant monotherapy as their first or second endocrine therapy were enrolled (group A). Patients with disease progression during fulvestrant monotherapy were subsequently registered in group B.

Eligible patients were enrolled at 55 investigation sites in academic and community settings in Japan, in accordance with the following key inclusion criteria: (1) women ≥ 20 but < 80 years old with histologically or cytologically confirmed metastatic HR-positive/human epidermal growth factor receptor 2 (HER2)-negative breast cancer; (2) treated with fulvestrant monotherapy as first- or second-line therapy for ABC; and (3) no previous systemic therapy for breast cancer (except for one line of cytotoxic chemotherapy). Patients who did not experience progressive disease during fulvestrant monotherapy and those with severe or uncontrolled medical conditions were excluded. This study was conducted according to the tenets of the Declaration of Helsinki and approved by the institutional review board of Fukushima medical university. All patients provided written informed consent prior to participation.

The study protocol was registered with the University Hospital Medical Information Network, Japan (protocol ID 000,016,109) and at Clinical trials.gov (NCT 02,376,985).

### Procedures

Fulvestrant (500 mg) was administered on the following days: 0, 14 (plus 7 days), 28 (plus 7 days), and every 28 (plus 7 days) days thereafter in two 5-mL intramuscular injections at each visit. Fulvestrant dose reductions were not permitted. Treatment continued until objective disease progression was noted or other criteria for discontinuation arose.

Study visits occurred during screenings (within 28 days before registration) and every 4 weeks thereafter until disease progression. Safety and tolerability were assessed at each visit.

Patients were subsequently registered in group B if they experienced disease progression during fulvestrant monotherapy. They continued receiving 500 mg fulvestrant via intramuscular injection in subsequent 28-day cycles and were also orally administered 125 mg palbociclib once daily for 3 weeks, followed by 1 week off in a 28-day cycle. The study treatment continued until disease progression, unacceptable toxic effects, consent withdrawal, or death. Dose interruption, reduction, or delay according to a predefined dose-modification strategy was acceptable for patients who experienced toxic effects related to the investigational drugs.

We assessed tumors at baseline and every 8 weeks (± 14 days) for the first 24 weeks after the first fulvestrant administration and every 12 weeks (± 14 days) thereafter using computed tomography, radiography, or both in all patients.

The assessment of adverse events included incidence and severity (graded according to the National Cancer Institute Common Terminology Criteria, Version 4.0), timing, seriousness, and relatedness to the study treatment.

Estrogen receptor-positive, progesterone receptor-positive tumors, or both, and HER2-negative tumors were locally assessed using an assay consistent with local standards.

### Outcome measurement

The primary objective was PFS from the start of combination therapy in group B patients (PFS1) (Supplemental Fig. 1). The secondary objective was PFS stratified by patients treated with fulvestrant monotherapy as the first- or second-line therapy in group A (PFS2). Additional endpoints included PFS from the start of monotherapy to failure combination therapy in group B (PFS3), time to treatment failure (TTF) from the start of both fulvestrant monotherapy and combination therapy, OS from the start of both fulvestrant monotherapy and combination therapy, objective response rates (fulvestrant monotherapy, combination of fulvestrant and palbociclib), safety (fulvestrant monotherapy, combination of fulvestrant and palbociclib), and biomarkers.

### Statistical analysis

We set the threshold median PFS of 5 months (null hypothesis), which was the reported difference between the fulvestrant monotherapy and combination therapy groups in the PALOMA-3 study. Assuming a median PFS1 of 8 months in group B, the minimum sample size required was 63 patients, with a power of 80% under a one-sided alpha of 0.05. The accrual and follow-up periods were set at 12 and 18 months, respectively. The target number of accrual patients was determined at 70 in the second registration (group B).

The primary analysis was the estimation of two-sided 90% confidence intervals (CIs) for PFS1 and hypothetical testing against the null hypothesis; the median was 5 months. The Kaplan–Meier method was used to summarize the PFS1-3, TTFs, and OS. The standard error of the annual rates and two-sided 90% CIs were calculated using Greenwood's formula. Frequencies and proportions were used to tabulate the overall response rates. The worst grades relating to safety observed during the treatment period were summarized.

## Results

### Patient characteristics

**Fig. 1:**
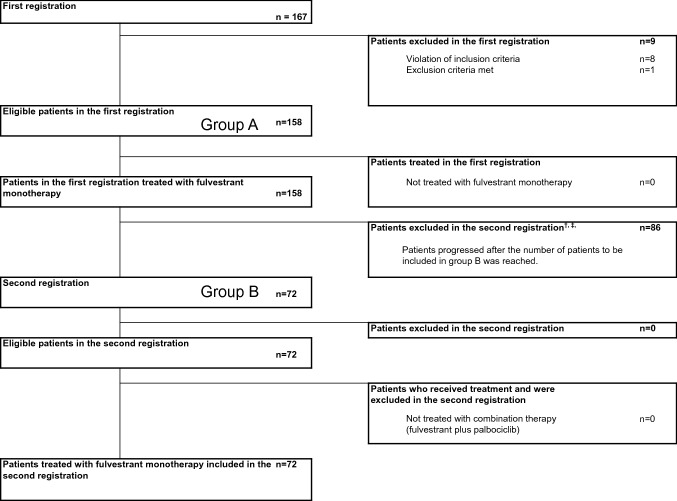
CONSORT Flow Diagram. ^†^patients with metastases to the central nervous system (including asymptomatic patients). ^‡^once 72 patients were enrolled in group B, enrollment was considered complete, and patients with disease progression in group A thereafter were not included

We enrolled 167 patients from 55 institutions between January 2018 and February 2020 in group A and 72 patients in group B. Nine patients from group A did not receive the protocol treatment and were excluded from the analysis. Once the 72 patients were enrolled in group B, enrollment was considered complete, and patients with disease progression in group A thereafter were not included (Fig. 1). Ultimately, the data of 158 patients were included in the analysis. The median follow-up time from enrollment was 23.8 and 8.9 months in groups A and B, respectively. Two patients who received monotherapy in group A were still receiving treatment at the data cutoff point.

In group B, the median age was 67 years; 20 (28%) patients did not receive ET for ABC, 52 (72%) patients received ET before fulvestrant monotherapy, 59 (82%) patients had progesterone receptor-positive tumors, and 82 (51%) patients had visceral disease (Table 1).

**Table 1:** Patient characteristics

No. of patients		Group A (FUL monotherapy)	Group B (combination therapy)
		158	72
Age (years)	Median (range)	67 (44–80)	67 (44–80)
BMI (kg/m^2^)	Mean (SD)	23.9 (4.0)	23.6 (3.9)
Previous treatment, *n* (%)			
Endocrine treatment	None	59 (37.3)	20 (27.8)
	≤ 1	99 (62.7)	52 (72.2)
Chemotherapy	None	142 (89.9)	65 (90.3)
	≤ 1	16 (10.1)	7 (9.7)
Distant metastasis, *n* (%)			
	De novo	46 (29.1)	18 (25.0)
	Metastatic	112 (70.9)	54 (75.0)
Nuclear grade, *n* (%)	I	42 (26.6)	18 (25.0)
	II	42 (26.6)	15 (20.8)
	III	15 (9.5)	8 (11.1)
	Unknown	59 (37.3)	31 (43.1)
Status of hormone receptor, *n* (%)			
ER	Positive	158 (100.0)	72 (100.0)
	Negative	0 (0.0)	0 (0.0)
PgR	Positive	126 (79.7)	58 (80.6)
	Negative	32 (20.3)	14 (19.4)
HER2	Positive	0 (0.0)	0 (0.0)
	Negative	158 (100.0)	72 (100.0)
Visceral metastasis, *n* (%)	Yes	82 (51.9)	38 (52.8)
	No	76 (48.1)	34 (47.2)
Adjuvant chemotherapy, *n* (%)	Yes	66 (41.8)	33 (45.8)
	No	41 (25.9)	18 (25.0)
Adjuvant endocrine therapy, *n* (%)	Yes	91 (57.6)	41 (56.9)
	No	16 (10.1)	10 (13.9)
Radiation therapy for the metastatic site, *n* (%)	Yes	31 (19.6)	11 (15.3)
	No	127 (80.4)	61 (84.7)

*FUL* fulvestrant; *BMI* body mass index; *SD* standard deviation; *ER* estrogen receptor; *PgR* progesterone receptor, *HER2* human epidermal growth factor receptor 2

### Clinical outcomes in group B

**Fig. 2:**
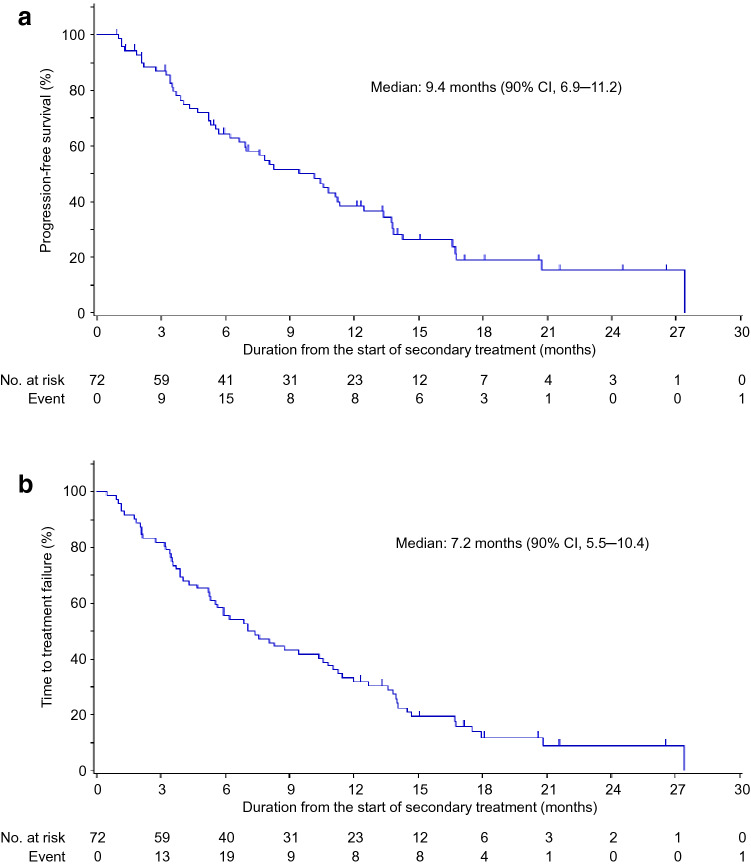
Data regarding the combination therapy group (Group B). **a** Progression-free survival (PFS1) and **b** time to treatment failure. *CI* confidence interval

**Fig. 3:**
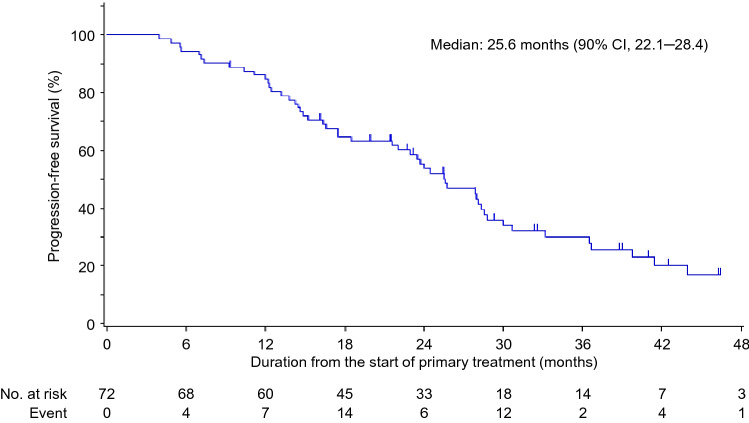
Progression-free survival from the start of monotherapy (PFS3). *CI* confidence interval

Regarding the primary endpoint, the median PFS1 in group B was 9.4 (90% CI = 6.9–11.2; *p* < 0.001) months (Fig. 2a). The TTF from the start of combination therapy in group B was 7.2 (90% CI = 5.5–10.4) months (Fig. 2b). The median PFS3 in group B was 25.6 (90% CI = 22.1–28.4) months (Fig. 3). PFS1 in group B patients who received fulvestrant as the first-line treatment (median, 8.2 months) was not as long as that of patients who received fulvestrant as the second-line treatment (median, 10.6 months) (Supplemental Fig. 2). PFS among patients with visceral disease (median, 11.3 months) was not significantly different from those without (median, 8.2 months). Other factors, such as de novo metastatic cancer and age > 65, were not significantly different regarding PFS.

In the *post-hoc* analysis, we explored whether patients who received longer-duration fulvestrant monotherapy would show longer PFS than those receiving shorter-duration monotherapy. The median PFS among patients with longer-duration monotherapy (> 1 year) was 3.7 months longer than that of patients with shorter-duration monotherapy (≤ 1 year) (11.3 vs. 7.6 months).

The overall response rate in group B was 8.3%, and the clinical benefit rate was 37.5% (Table 2).

**Table 2:** Overall response rates

No. of patients		Group A (FUL monotherapy)	Group B (combination therapy)
		158	72
Best overall response, *n* (%)	CR	5 (3.2)	1 (1.4)
	PR	30 (19.0)	5 (6.9)
	SD or non-CR/non-PD	92 (58.2)	54 (75.0)
	PD	28 (17.7)	6 (8.3)
	NE	1 (0.6)	6 (8.3)
	Loss	2 (1.3)	–
Overall response rate^a^, n (%) [95% CI]		35 (22.4) [17.1, 28.6]	6 (8.3) [3.7, 15.8]
CR + PR + SD for more than 6 months^a^, n (%) [95% CI]		92 (59.0) [52.1, 65.6]	27 (37.5) [28.0, 47.8]

*FUL* fulvestrant; *CR* complete response rate; *PR* partial response rate; *SD* stable disease; *PD* progressive disease; *NE* not evaluable; *CI* confidence interval; *RECIST* Response Evaluation Criteria in Solid Tumors

^a^the denominator represents the number of patients with missing data on the RECIST evaluation

### Clinical outcomes in group A

**Fig. 4:**
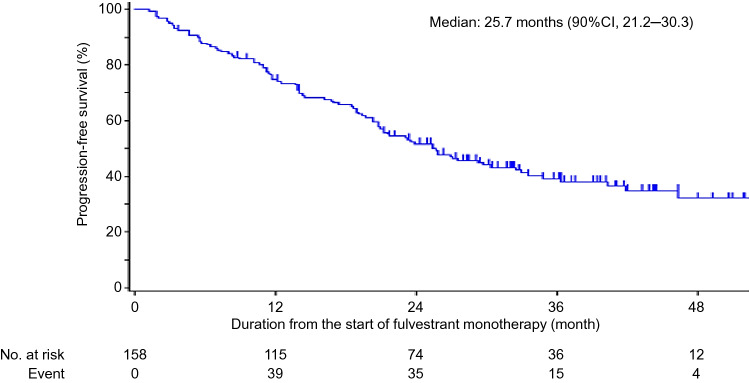
Progression-free survival (PFS2) in Group A (fulvestrant monotherapy). *CI* confidence interval

The median PFS2 for monotherapy patients in group A was 25.7 (90% CI = 21.2–30.3) months (Fig. 4). PFS for patients in group A who received fulvestrant as the first-line treatment (median, 30.3 months) was not significantly different from that of patients who received fulvestrant as the second-line treatment (median, 21.5 months). PFS for patients in group A with visceral disease (median, 25.8 months) was not significantly different from those without (median, 25.4 months). The overall response rate in group A was 22.4%, and the clinical benefit rate was 58.9% (Table 2).

### Safety

The adverse events are shown in Tables 3 and 4, with no new safety signals detected. Ultimately, 65 (90%) patients in group B and 78 (49%) in group A reported the occurrence of at least one adverse event in one or more cycles. Hematological toxicities were the most common adverse events in group B. Neutropenia occurred in 62 (86%) patients in group B (43 [59%]) and 17 [23%] were grades 3 and 4, respectively). Infections and febrile neutropenia occurred in two patients. Nine (12.5%) patients reported at least one serious treatment-related adverse event.

**Table 3:** Adverse events

N (%)	Group A (FUL monotherapy)	Group B (combination therapy)
No. of patients	158	72
All AEs	78 (49.4)	65 (90.3)
Treatment-related AEs^a^	73 (46.2)	65 (90.3)
Grade 3 or 4 AEs	71 (44.9)	64 (88.9)
Treatment-related grade 3 or 4 AEs	66 (41.8)	64 (88.9)
All-cause death related to AEs^b^	0 (0.0)	0 (0.0)
Treatment-related deaths^b^	0 (0.0)	0 (0.0)
Serious AEs	14 (8.9)	9 (12.5)
AEs leading to withdrawal from treatment^d^	5 (3.2)	5 (6.9)

*FUL* fulvestrant; *AEs* adverse events

^a^cases in which the AEs were related to fulvestrant or palbociclib

^b^the outcome was “death”

^b^cases in which ^a^ and ^b^ apply

^d^discontinuation of either fulvestrant or palbociclib

**Table 4:** Adverse events by grade in the combination therapy group

*N* (%)	Total	Grade 1^a^	Grade 2^a^	Grade 3^a^	Grade 4^a^	Grade 5^a^
No. of patients	72	–	–	–	–	–
All AEs	65 (90.3)	24 (33.3)	27 (37.5)	63 (87.5)	17 (23.6)	0 (0.0)
Decreased neutrophil count	62 (86.1)	0 (0.0)	2 (2.8)	43 (59.7)	17 (23.6)	0 (0.0)
Decreased white blood cell	50 (69.4)	0 (0.0)	13 (18.1)	36 (50.0)	1 (1.4)	0 (0.0)
Decreased hemoglobin	25 (34.7)	12 (16.7)	10 (13.9)	3 (4.2)	0 (0.0)	0 (0.0)
Decreased platelet count	21 (29.2)	15 (20.8)	4 (5.6)	2 (2.8)	0 (0.0)	0 (0.0)
Mucositis oral	14 (19.4)	7 (9.7)	5 (6.9)	2 (2.8)	0 (0.0)	0 (0.0)
Alopecia	6 (8.3)	5 (6.9)	1 (1.4)	0 (0.0)	0 (0.0)	0 (0.0)
Infection	4 (5.6)	0 (0.0)	0 (0.0)	4 (5.6)	0 (0.0)	0 (0.0)
Osteonecrosis of jaw	3 (4.2)	0 (0.0)	0 (0.0)	3 (4.2)	0 (0.0)	0 (0.0)
Anorexia	2 (2.8)	0 (0.0)	0 (0.0)	2 (2.8)	0 (0.0)	0 (0.0)
Febrile neutropenia	2 (2.8)	0 (0.0)	0 (0.0)	2 (2.8)	0 (0.0)	0 (0.0)
Pain	2 (2.8)	0 (0.0)	0 (0.0)	2 (2.8)	0 (0.0)	0 (0.0)
Pneumonitis	1 (1.4)	0 (0.0)	1 (1.4)	0 (0.0)	0 (0.0)	0 (0.0)
Increased alanine aminotransferase	1 (1.4)	0 (0.0)	0 (0.0)	1 (1.4)	0 (0.0)	0 (0.0)
Dizziness	1 (1.4)	0 (0.0)	0 (0.0)	1 (1.4)	0 (0.0)	0 (0.0)
Lower gastrointestinal hemorrhage	1 (1.4)	0 (0.0)	0 (0.0)	1 (1.4)	0 (0.0)	0 (0.0)
Hepatobiliary disorders and liver dysfunction	1 (1.4)	0 (0.0)	0 (0.0)	1 (1.4)	0 (0.0)	0 (0.0)
Pleural effusion	1 (1.4)	0 (0.0)	0 (0.0)	1 (1.4)	0 (0.0)	0 (0.0)
Thromboembolic event	1 (1.4)	0 (0.0)	0 (0.0)	1 (1.4)	0 (0.0)	0 (0.0)
Malaise	1 (1.4)	0 (0.0)	0 (0.0)	1 (1.4)	0 (0.0)	0 (0.0)
Respiratory, thoracic, and mediastinal disorders and interstitial pneumonia	1 (1.4)	0 (0.0)	0 (0.0)	1 (1.4)	0 (0.0)	0 (0.0)
Periodontal disease	1 (1.4)	0 (0.0)	0 (0.0)	1 (1.4)	0 (0.0)	0 (0.0)
Bullous dermatitis	1 (1.4)	0 (0.0)	0 (0.0)	1 (1.4)	0 (0.0)	0 (0.0)
Vomiting	1 (1.4)	0 (0.0)	0 (0.0)	1 (1.4)	0 (0.0)	0 (0.0)

^a^Common terminology criteria for adverse events v4.0-Japan Clinical Oncology Group

Dose reductions occurred in 53 patients (73%) in group B, predominantly due to hematological toxicity (46 [86%]). Similarly, dose delays occurred in 63 (87%) patients. The final palbociclib dose before disease progression was 125 mg in 18 (25%) patients, 100 mg in 24 (33.8%) patients, 75 mg in 14 (19%) patients, and 75 mg in 10 (14%) patients with 2 weeks on and 2 weeks off.

## Discussion

This study investigated the strategy of adding palbociclib to fulvestrant after disease progression in patients with HR-positive/HER2-negative ABC. Our data suggest that this treatment strategy is safe, with a median PFS1 of 9.4 months. The primary endpoint was met based on study assumptions. Palbociclib may reverse endocrine therapy resistance in patients receiving fulvestrant treatment. The median PFS3 (fulvestrant monotherapy to treatment failure with fulvestrant plus palbociclib) was 25.6 months. These data are similar to those of combined ET with CDK 4/6 agents in frontline treatment. The strategy of adding palbociclib to ET was also investigated in the TREnd trial [9]. Regarding the activity of palbociclib when given as either a single-agent or in combination with ET after disease progression, the clinical benefit rates for the single-agent and combination groups were 60% and 54%, respectively. The median PFS was 6.5 months and 10.8 months in the single-agent and combination groups, respectively. The strategy of adding palbociclib appears to be beneficial and safe for patients with ET resistance.

Endocrine therapy remains the standard HR-positive/HER2-negative ABC therapy before chemotherapy [4]. Recently, multiple-target therapy with ET was approved, including CDK 4/6, mammalian/mechanistic target of rapamycin, and phosphoinositide 3-kinase inhibitors. However, ET is typically limited to selective estrogen receptor modulators, selective estrogen receptor degraders, and AIs. Most patients receiving targeted therapy have resistance to the same class of ET; therefore, strategies to overcome resistance are of ongoing interest. Preclinical evidence suggests that palbociclib could overcome conditioned resistance to a given ET [11].

Multiple large, phase III studies have confirmed the efficacy of ET combined with CDK 4/6 agents in front- or later-line treatment settings [7, 8, 12, 13]. The strategy of changing to another endocrine agent combined with a CDK4/6 inhibitor has been explored in large phase III clinical trials, with superiority observed in combined therapy over ET alone [7, 8, 12, 13]. The combination of fulvestrant with CDK 4/6 agents in frontline treatment prolonged PFS and OS compared with fulvestrant monotherapy in the MONARCH 2 and MONALEESA-3 trials [13] [14]. The median PFS in patients receiving fulvestrant combined with a CDK4/6 agent in a first-line setting was 33.6 months in the MONALEESA-3 trial, 14.6 months in a second-line setting in the MONALEESA-3 trial, and 16 months in the MONARCH-2 trial. The PFS3 from fulvestrant monotherapy to combined fulvestrant and palbociclib therapy was 25.6 months. However, our strategy included patients who experienced disease progression twice. Despite the limitations in making indirect comparisons across studies, our data and those of the TRrend trial suggest that the strategy of adding a CDK 4/6 inhibitor to the same ET after disease progression may merit further study in a selected population with prolonged benefit during their prior line of ET.

Another noteworthy study is the SONIA-trial [15], an investigator-initiated, multicenter, randomized Phase III study. Its primary objective is to evaluate if treatment with a non-steroidal AI combined with CDK4/6 inhibition as the 1st-line therapy followed by fulvestrant as the 2nd-line therapy (strategy A) could improve PFS, as compared to treatment with a non-steroidal AI as the 1st-line therapy followed by fulvestrant combined with CDK4/6 inhibition as the 2nd-line therapy (strategy B). The SONIA trial will provide evidence in terms of benefit in PFS with an up-front CDK4/6 inhibitor in combination with ET.

In the FALCON trial, fulvestrant had a beneficial treatment effect compared with that of anastrozole in patients with de novo stage 4 cancer, particularly in patients without visceral disease [3]. However, the PARSIFAL study showed that the combination of fulvestrant and palbociclib showed no superiority in PFS over letrozole-palbociclib combination therapy in a patient population among patients with previously untreated ABC [16]. Subgroup analysis showed that patients with de novo stage 4 and non-visceral disease showed no benefit from fulvestrant combined with palbociclib, which is inconsistent with the results of the FALCON trial. Combination therapy with CDK 4/6 inhibitors did not maintain fulvestrant efficacy compared with an AI.

Our data indicated a correlation between fulvestrant monotherapy and treatment duration after combining palbociclib with fulvestrant. In the TREnd trial, subgroup analysis of patients with a history of ET ≥ 6 months showed better PFS than patients with a history of ET < 6 months. Our trial and the TREnd trial demonstrated that patients with a history of long-term ET had better PFS.

Acquired resistance to AI frequently stems from *ESR1*-mutated subclones, which may be sensitive to fulvestrant and oral selective ER degraders (SERDs). The PADA-1 trial [17] showed that the combination of fulvestrant with palbociclib prolonged PFS in patients with rising *ESR1* mutations detected by circulating tumor (ct)DNA*.* In addition, the SELENA-6 trial will evaluate the hypothesis that switching to a SERD combination with a CDK4/6 inhibitor is effective for patients with detectable *ESR1* mutations but without disease progression during 1st-line treatment with AI and a CDK4/6 inhibitor. Oral SERDs such as Elacestrant [18] have demonstrated a significant PFS improvement in patients with *ESR1* mutations. Our study collected plasma samples at three time points: before adding palbociclib, on day 15 of the first cycle, and at the end of treatment. Analysis of ctDNA helped us select our treatment strategy, and palbociclib may have the potential to reverse endocrine therapy resistance in patients resistant to fulvestrant.

This study had some limitations. First, the number of patients was too small to confirm whether patients overcame ET resistance. Second, in the single-arm registration study of fulvestrant monotherapy, the study population was more fulvestrant sensitive than the general population. Moreover, patients with early treatment failure on fulvestrant monotherapy were difficult to register. Third, the median follow-up duration was too short to evaluate OS in patients with HR-positive ABC.

In conclusion, our data suggest that palbociclib plus fulvestrant after disease progression with fulvestrant monotherapy may be effective and safe in patients with HR-positive/HER2-negative ABC or MBC. This strategy may be an option for patients with ABC resistant to fulvestrant monotherapy.

## Supplementary Information

Below is the link to the electronic supplementary material.

Supplementary file1 (PDF 295 KB)

## Data Availability

The datasets generated and/or analyzed during the current study are available from the corresponding author upon reasonable request.
