# Supplementary material for: Fulvestrant plus palbociclib in advanced or metastatic hormone receptor-positive/human epidermal growth factor receptor 2-negative breast cancer after fulvestrant monotherapy: Japan Breast Cancer Research Group-M07 (FUTURE trial)
Source: Breast Cancer Res Treat. 2023 Mar 31;199(2):253–63. doi: 10.1007/s10549-023-06911-5 (PMC10175424; doi:10.1007/s10549-023-06911-5)
Supplement: Supplementary file 1 — Supplementary file1 (PDF 295 KB) [file 10549_2023_6911_MOESM1_ESM.pdf]

Supplemental
Figure 1 Study Design

Study Schema

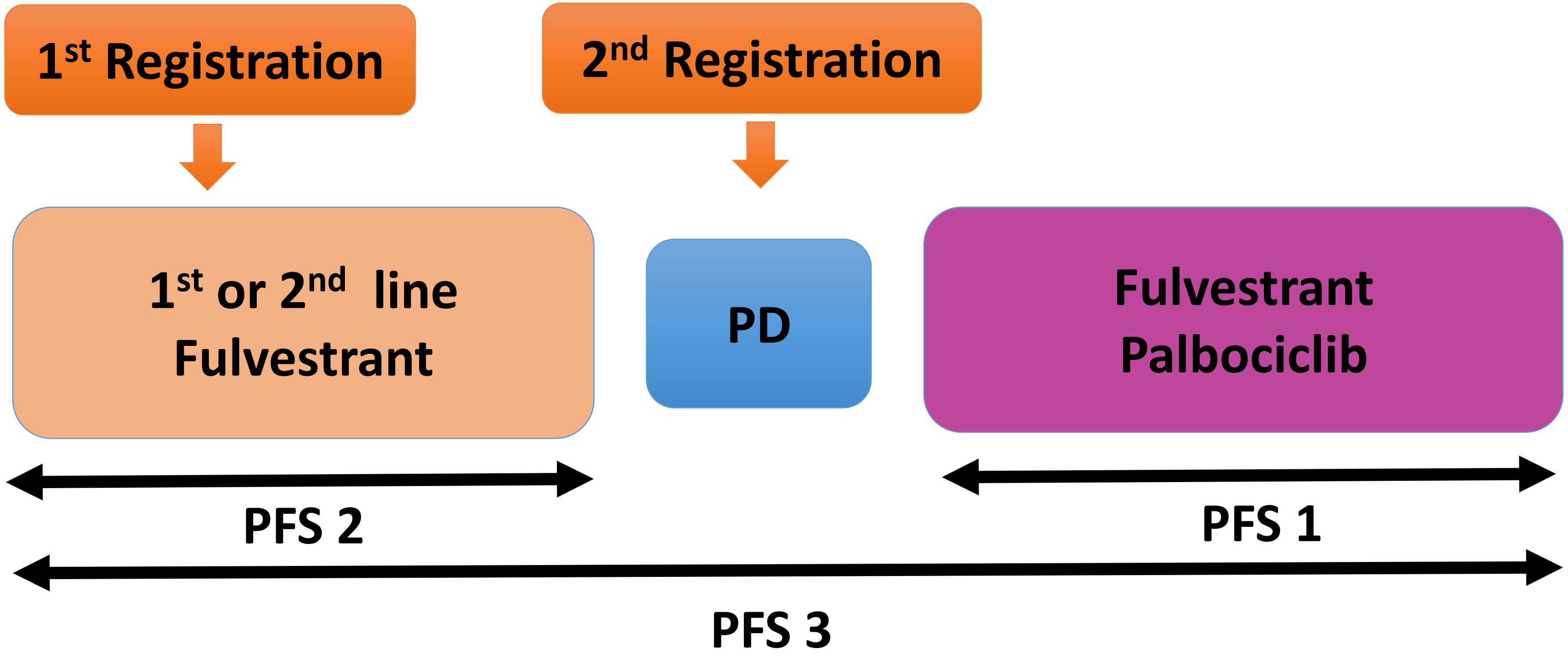

Supplemental

Figure 2(a) Progression-free survival in the second registration (combination therapy) by primary treatment duration

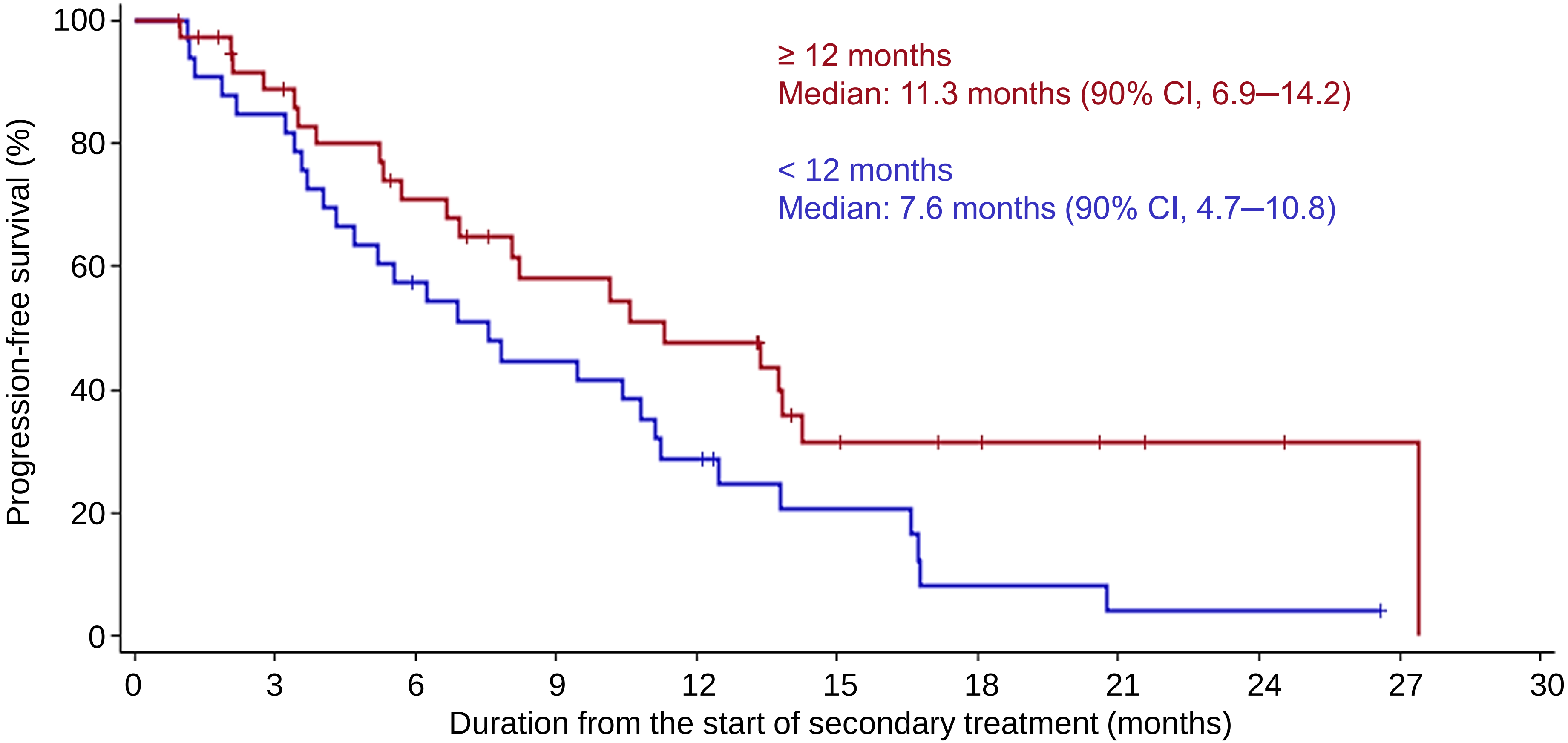

**12 months and longer**											
No. at risk	39	31	23	17	14	7	5	3	2	1	0
Event	0	4	6	4	3	4	0	0	0	0	1
**Less than 12 months**											
No. at risk	33	28	18	14	9	5	2	1	1	0	0
Event	0	5	9	4	5	2	3	1	0	0	0

Supplemental

Figure 2(b) Progression-free survival by primary and secondary fulvestrant therapy

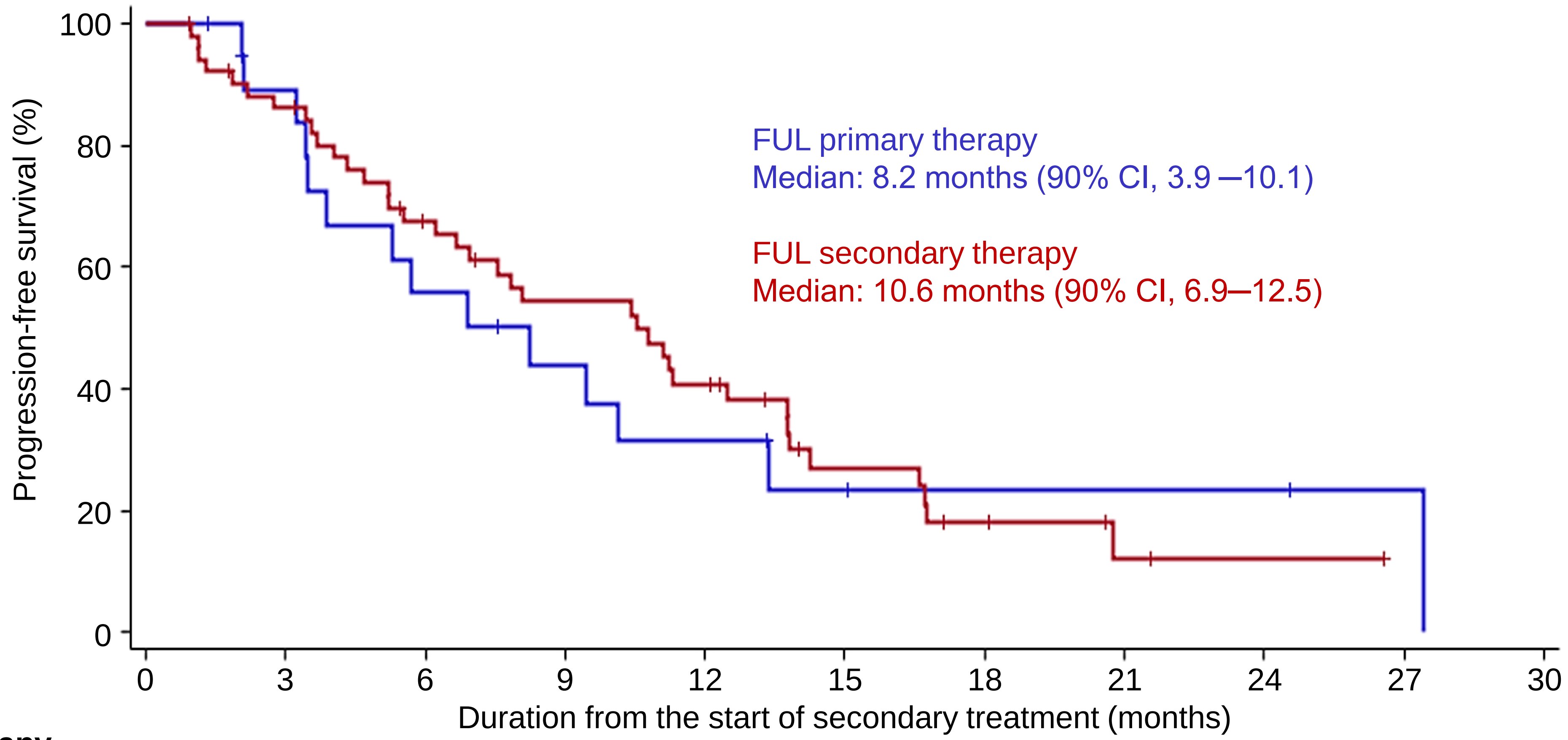

**FUL primary therapy**											
No. at risk	20	16	10	7	5	3	2	2	2	1	0
Event	0	2	6	2	2	1	0	0	0	0	1
**FUL secondary therapy**											
No. at risk	52	43	31	24	18	9	5	2	1	0	0
Event	0	7	9	6	6	5	3	1	0	0	0

Supplemental

Figure 2(c) Progression-free survival in second registration (combination therapy) by visceral metastasis

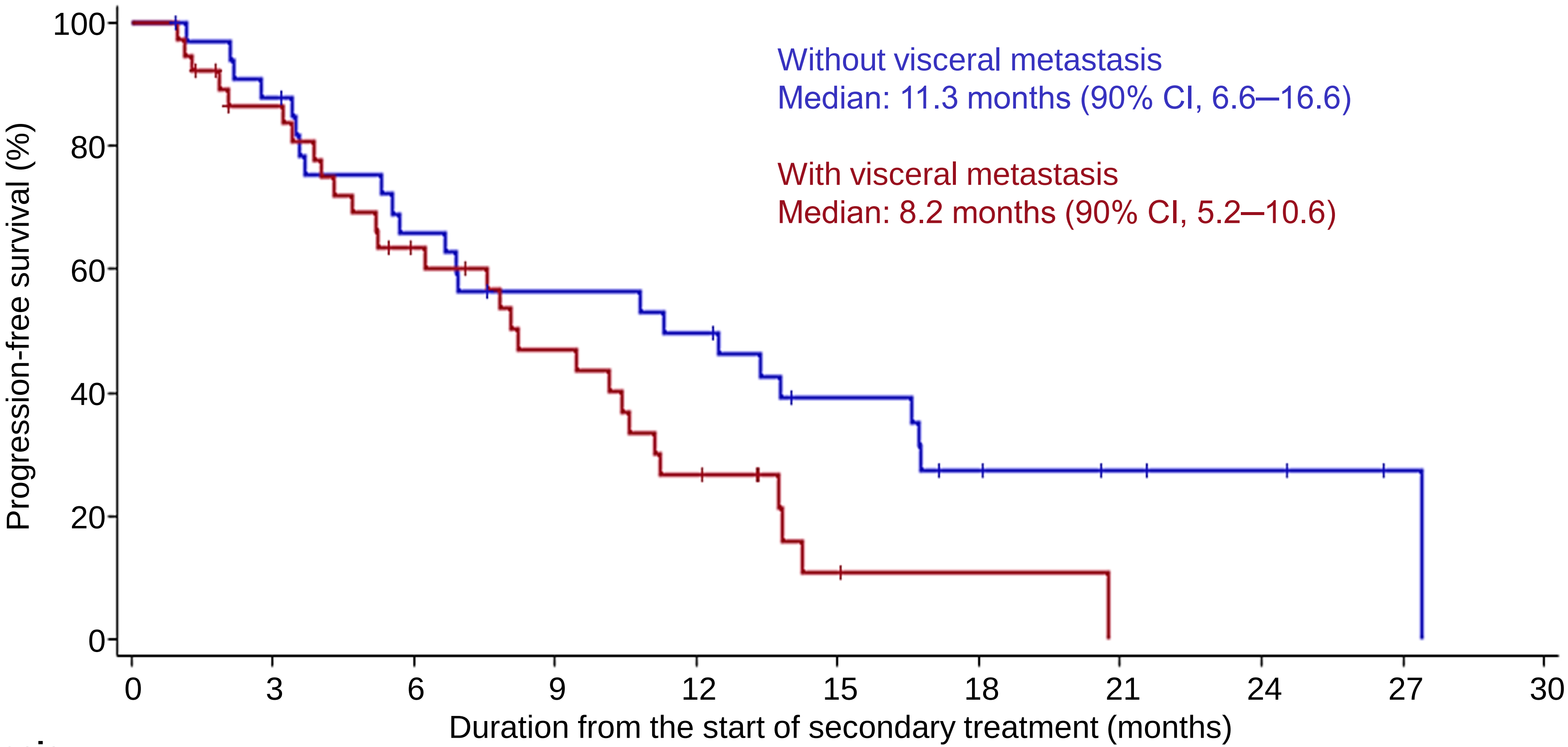

**Without metastasis**											
No. at risk	34	29	21	17	15	10	6	4	3	1	0
Event	0	4	7	3	2	3	3	0	0	0	1
**With metastasis**											
No. at risk	38	30	20	14	8	2	1	0	0	0	0
Event	0	5	8	5	6	3	0	1	0	0	0

Supplemental

Figure 2(d) Comparison of progression-free survival in second-registration (combination therapy) between *de novo* stage Ⅳ and metastatic cancer

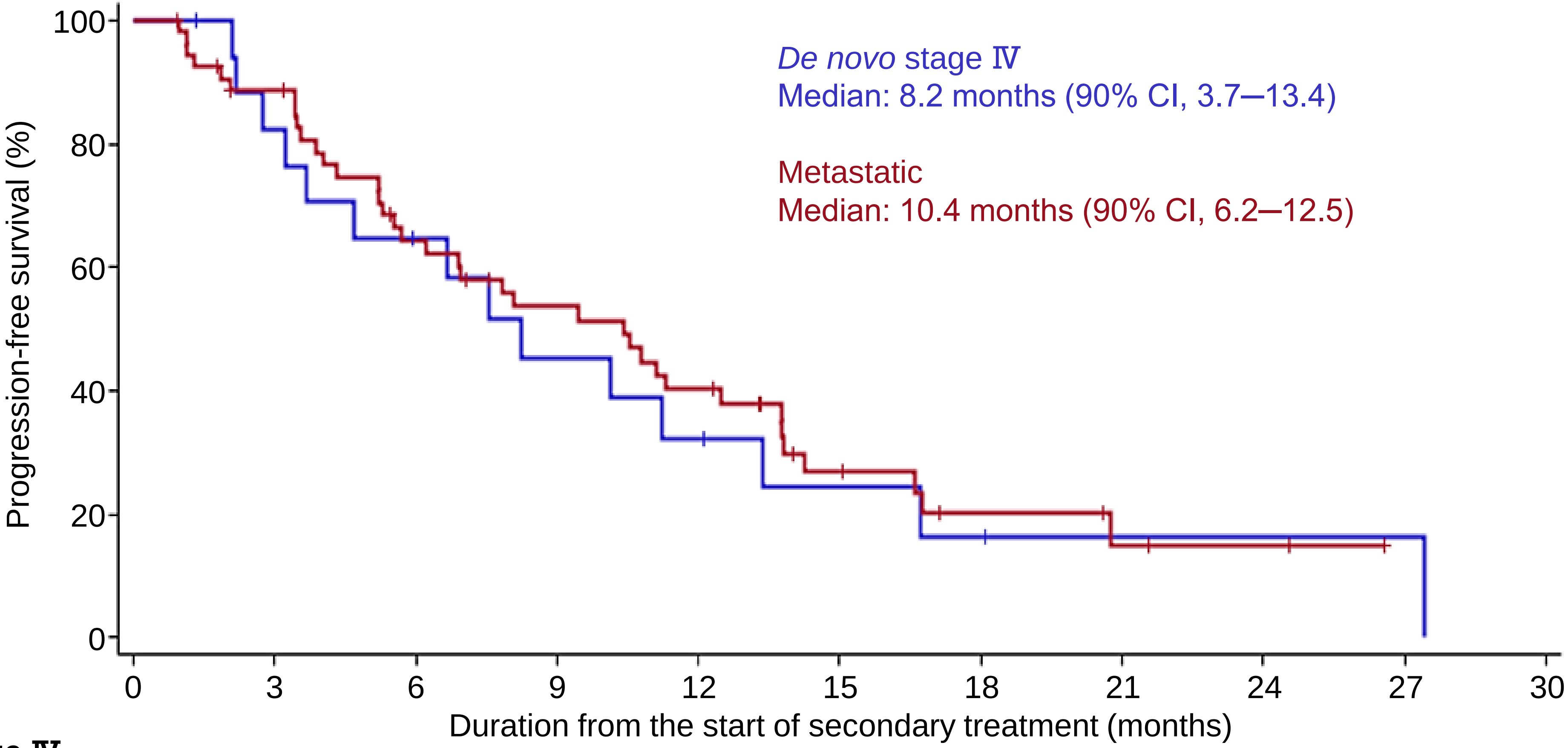

***De novo* stage Ⅳ**											
No. at risk	18	14	10	7	5	3	2	1	1	1	0
Event	0	3	3	3	2	1	1	0	0	0	1
**Metastatic**											
No. at risk	54	45	31	24	18	9	5	3	2	0	0
Event	0	6	12	5	6	5	2	1	0	0	0
